# Self-Management Group Exercise Extends Healthy Life Expectancy in Frail Community-Dwelling Older Adults

**DOI:** 10.3390/ijerph14050531

**Published:** 2017-05-15

**Authors:** Minoru Yamada, Hidenori Arai

**Affiliations:** 1 Graduate School of Comprehensive Human Sciences, University of Tsukuba, 3-29-1 Otsuka, Bunkyo-ku, Tokyo 112-0012, Japan; 2 National Center for Geriatrics and Gerontology, 7-430, Morioka-cho, Obu, Aichi 474-8511, Japan; harai@kuhp.kyoto-u.ac.jp

**Keywords:** self-management group, long-term care insurance, disability

## Abstract

Preventing frailty and its adverse health outcomes is crucial in countries with a large elderly population, such as Japan. Since the long-term care insurance (LTCI) system was launched, the number of certified older adults with LTCI service requirement has continued to increase. This is a serious problem, because the LTCI service requirement certification is equivalent to disability. The aim of this study was to evaluate the effect of a self-management group intervention on new LTCI service requirement certifications in community-dwelling older adults in Japan. We analyzed the cohort data from a prospective study. In this study, we recruited community-dwelling adults aged 65 years and older who were independent in a city in Kyoto prefecture in 2012. The subjects in the participation group (n = 1620) attended 60-min group training sessions once or twice every two weeks from December 2012 to December 2016. The exercise sessions consisted of mild-intensity aerobic exercise, mild strength training, flexibility and balance exercises, and cool-down activities. These exercise classes were facilitated by well-trained volunteer staff. The outcome measure was the number of new LTCI requirement certifications during a four-year follow-up period. During the four-year follow-up period, 247 subjects (15.2%) in the participation group and 334 (20.6%) in the control group were newly certified for LTCI service requirements. The hazard ratio for new LTCI service requirements in the participation group compared with the control group was 0.73 (95% CI = 0.62–0.86) in the four-year follow-up period. These results indicate the usefulness of self-management group exercise to reduce the incidence of disability in older adults. Thus, increasing self-management group activities in each community should be encouraged.

## 1. Introduction

Frailty is a risk factor for disability, hospitalization and mortality [1,2,3,4]. The prevalence of frailty is approximately 10% in the community-dwelling older population [2,4,5], and this high prevalence is a crucial social issue in countries with large elderly populations, such as Japan. Therefore, preventing frailty and its adverse health outcomes is critical in such countries.

In 2000, the Japanese government launched the long-term care insurance (LTCI) system. This system provides friendly services to older adults who are certified as requiring support or care according to their level of frailty or disabilities [6]. However, since the LTCI system was launched, the number of certified disabled older adults has continued to increase in Japan. The increasingly disabled older population has led to increasing social security expenses, which is now a major issue in Japan.

In 2006, the Japanese government revised the LTCI system, and new preventive benefits were introduced. The Japanese LTCI system has focused on preventive care services for older adults at risk for needing care in the near future, according to this revision. Japanese local governments sought to identify frail older adults by using the Kihon checklist, which was distributed via a mail survey [7]. Each Japanese local government system provides several care preventive services to aid in healthcare professionals’ disability prevention efforts in frail older adults. Intriguingly, the effect of disability prevention programs on frail older adults has been relatively significant, and these programs have prevented disability in frail older adults [8]. However, there have been major issues with these care preventive services, which too few participants attended. 

The Japanese government has recently encouraged local governments to increase the number of self-management exercise groups without health care professional supervision for community-dwelling older adults. However, the effect of this self-management group intervention on the incidence of disability remains unclear. The aim of this study, therefore, was to evaluate the effects of the self-management group intervention on disability in community-dwelling older adults. We hypothesized that subjects who attended the self-management group would have a lower incidence of new LTCI service requirement certification than non-participants.

## 2. Methods

### 2.1. Participants

We analyzed the cohort data from a prospective study. In this study, we recruited community-dwelling adults aged 65 years and older who were living independently in a city in Kyoto prefecture in 2012. The exclusion criteria were older adults who had already been designated as being activities of daily living (ADL)-dependent and who were already eligible to receive the benefits of the LTCI services.

A total of 18,562 residents were eligible for this study in December 2012. The self-administered Kihon checklist was mailed to 18,562 subjects, and the response rate was 85.7%. We further excluded individuals who moved out of the city during the four-year follow-up period, thus leaving a total of 15,901 participants. One thousand six hundred and twenty (10.2%) older adults participated in self-management group activities. This study was conducted in accordance with the guidelines proposed by the Declaration of Helsinki, and the study protocol was reviewed and approved by the Ethics Committee of Tsukuba University Graduate School of Comprehensive Human Sciences (28-88).

#### Kihon Checklist

The Kihon checklist is a frailty assessment tool, which was developed by the Japanese Ministry of Health, Labor and Welfare [7]. The Kihon checklist consists of simple 25 questions (with yes/no responses) that are divided into seven domains: lifestyle (questions 1 to 5), motor abilities (questions 6 to 10), nutrition (questions 11 to 12), oral functions (questions 13 to 15), seclusion (questions 16 to 17), forgetfulness (questions 18 to 20), and emotions (questions 21 to 25). We defined scores of 8 or more as frail, 4 to 7 as pre-frail, and 0 to 3 as robust, according to Satake’s criteria [9].

### 2.2. Self-Management Group

The local government has made efforts to increase the number of self-management groups since 2012. In December 2016, 106 groups were established in the city, and 1620 older adults participated in self-management group activities.

The activities were composed of 60 min of group training sessions once or twice every two weeks from December 2012 to December 2016. The exercise classes were facilitated by well-trained volunteer staff. The exercise sessions were completed according to a standardized format consisting of 10 min of light-intensity aerobic exercise, 20 min of mild strength training, 20 min of flexibility and balance exercises, and 10 min of cool-down activities. The aerobic exercise comprised global movement of the legs, trunk, and arms involving all joints and major muscle groups. Strength training consisted of progressive resistive exercises using a person’s own body weight. 

### 2.3. Education of the Volunteer Staff

Before the intervention, the volunteer staff members were invited to the local government office, where a physiotherapist and public health nurse provided over 16 hours of instruction about the evidence supporting the benefits of exercise, how to exercise, and the management of health risk. In addition, the volunteer staff received follow-up lectures about any updated information on exercise and risk management from a physiotherapist and public health nurse once every year.

### 2.4. Propensity Score Matching

We used propensity score matching to create a matched control group such that the control and study groups were well matched on the basis of all measured baseline characteristics such as age, gender, body mass index (BMI), and each item on the Kihon checklist. We estimated the scores of the participation group for each subject by using a multivariable logistic regression model. We were able to match 1620 pairs of intervention and control subjects who had similar propensity scores.

### 2.5. Outcome Measures

The outcome measure was the new LTCI service requirement certification over a two- or four-year follow-up period. 

### 2.6. Statistical Analysis

Baseline characteristics of the participation and control groups were compared to examine the comparability of the two groups. Differences in the demographic variables between the groups were analyzed using Student’s *t*-test or chi-square test. 

Kaplan-Meier survival curves were calculated for the group newly determined to need LTCI services. Cox proportional hazards models were used to estimate the hazard ratios (HR) and 95% confidence intervals (CI) of the relationships between groups and the time to new LTCI service requirement certifications. Survival time was defined as the time between enrollment and either a new LTCI service requirement certification two years after study entry (31 December 2014) or four years after study entry (31 December 2016). For stratified analysis according to the level of frailty, we divided the cohort into three groups; robust, pre-frail, and frail, according to the Kihon checklist. 

The data were analyzed using SPSS software (Statistical Package for the Social Sciences, version 21.0; SPSS, Inc., Chicago, IL, USA). A *p* value <0.05 was considered to be statistically significant for all of the analyses.

## 3. Results

The total number of participants in the participation group was 1620, and we therefore selected 1620 matched controls for propensity matching. Subjects in the intervention and control groups were comparable and well matched with regard to their baseline characteristics (Table 1). In each group, 420 older adults (25.9%) had a Kihon checklist of 8 points or greater (frail), 591 older adults (36.5%) had a Kihon checklist of 4 to 7 points (pre-frail), and 609 older adults (37.6%) had a Kihon checklist of 0 to 3 points (robust). In the participation group, no severe health problems, such as cardiovascular or musculoskeletal complications, occurred during the exercise sessions. Minor problems reported included muscle ache and fatigue. All problems were managed easily by the adjustment of the participation program and were improved during the intervention. In the four-year follow-up period, 13 subjects (0.8%) in the participation group and 55 (3.4%) in the control group died.

**Figure 1:**
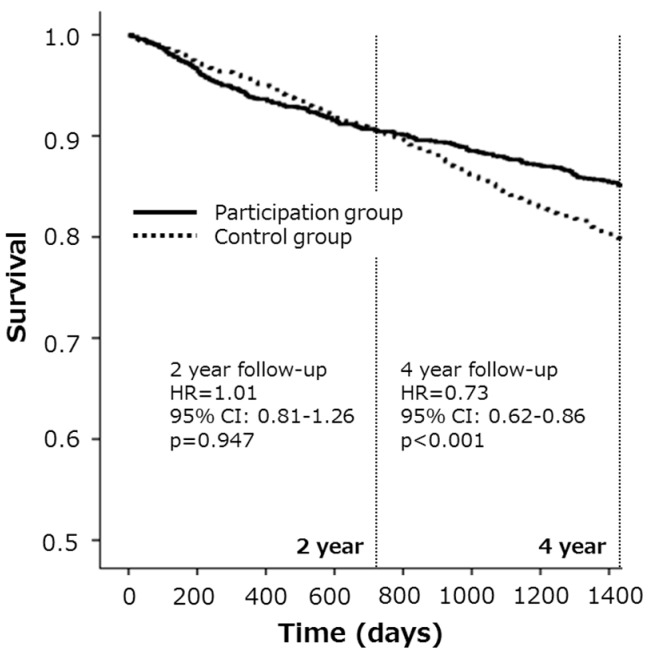
Kaplan-Meier survival curves for new LTCI service requirements are presented to compare the disability incidence in the total cohort. During the two-year follow-up period, 9.6% of participants in the participation group and 9.6% of the participants in the control group were newly certified for LTCI service requirements. Similarly, during the four-year follow-up period, 15.2% in the participation group and 20.6% in the control group were newly certified for long-term care insurance (LTCI) service requirements. During the four-year follow-up period but not the two-year follow-up period, the participation group, compared with the control group, had a significantly reduced risk for LTCI service needs (hazard ratios (HR) = 0.73, 95% CI = 0.62–0.86).

Overall, during the two-year follow-up period, 155 subjects (9.6%) in the participation group and 155 (9.6%) in the control group were newly certified for LTCI service requirements. Similarly, during the four-year follow-up period, 247 subjects (15.2%) in the participation group and 334 (20.6%) in the control group were newly certified for LTCI service requirements. The hazard ratio for new LTCI service requirements in the participation group compared with the control group in the two- and four-year follow-up periods was 1.01 (95% CI = 0.81–1.26) and 0.73 (95% CI = 0.62–0.86), respectively (Table 2, Figure 1).

**Figure 2:**
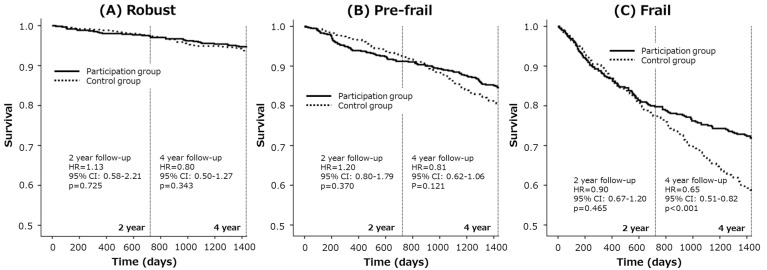
Kaplan-Meier survival curves for new LTCI service requirements are presented to compare the disability incidence in robust (**A**), pre-frail (**B**), and frail older adults (**C**). In the frail older adults but not in the robust or pre-frail older adults, the participation group had a significantly lower risk of needing LTCI services than the control group. In the frail older adults, the hazard ratios for new LTCI service requirements in the participation group compared with the control group in two- and four-year follow-up periods were 0.90 (95% CI = 0.67–1.20) and 0.65 (95% CI = 0.51–0.82), respectively.

We next performed sub-group analysis in the robust, pre-frail and frail older adults. In the robust older adults, the hazard ratios for new LTCI service requirement in the participation group compared with the control group in two-year and four-year follow-up periods were 1.13 (95% CI = 0.58–2.21) and 0.80 (95% CI = 0.50–1.27), respectively (Table 2, Figure 2). In the pre-frail older adults, these numbers were 1.20 (95% CI = 0.80–1.79) and 0.81 (95% CI = 0.62–1.06), respectively (Table 2, Figure 2), and in the frail older adults, they were 0.90 (95% CI = 0.67–1.20) and 0.65 (95% CI = 0.51–0.82), respectively (Table 2, Figure 2).

## 4. Discussion

In this study, we found that community-based self-management activities effectively reduced new LTCI service requirement certifications during a four-year follow-up period in community-dwelling older adults. Intriguingly, frail older adults, but not robust or pre-frail older adults, showed a significant reduction in their new LTCI service requirement certifications. In addition, we found that the effect was apparent after four years but was not apparent after only two years. Thus, this type of participation program was found to be useful in preventing new LTCI service requirement certifications during a relatively long-term follow-up period in frail older adults.

Physical exercise has a very important role in maintaining and improving health conditions in older adults. In particular, it promotes muscle function, which is helpful to prevent declines in the ability to complete activities of daily living [10]. A recent meta-analysis has shown that a sufficient amount of resistance training, even light load intensity training, leads to improvements in muscle function in older adults [11]. In addition, to maintain or improve muscle function, older adults must continue their physical exercise. Several reports have indicated that even if the muscle function is improved by resistance training, it will gradually decline during the detraining period in older adults [12,13,14,15]. Thus, there is a possibility that the physical function was maintained or improved by consecutive mild physical exercise in older adults who attended the community-based self-management group.

In our study, only frail older adults, but not robust or pre-frail older adults, showed a significantly lower incidence of new LTCI service requirement certifications. This result may have been because the load intensity of training was too light to affect the robust or pre-frail older adults. In addition, many frail older adults carry out less physical activity than robust older adults [16], such that joining the community-based self-management group led to the most dramatic improvement in the physical activity levels of the frail older adults. Furthermore, we believe that a lifestyle change occurred in subjects who attend the self-management exercise group for at least two years.

Attending physical exercise classes, joining a community salon, and participating in various social activities are very effective in preventing new LTCI service requirement certifications [17,18]. In particular, social participation is a robust promoter of healthy life expectancy. Thus, joining the community-based self-management group may not only lead to physical improvements but may also promote social activity in older adults. The community-based self-management group includes elements of physical exercise and social participation, both of which are believed to be effective in preventing disability.

There were several limitations of this study that warrant mention. First, we used a longitudinal observation study design. Ideally, a randomized controlled trial should be used to confirm the present results. Second, we did not measure physical performance, such as walking speed, strength or balance ability. Therefore, we were not able to evaluate the specific physical effects of this self-management group intervention on the community-dwelling older adults. Third, we did not analyze visit records. Therefore, we were not able to consider the effects on a lower incidence of disability in terms of adherence to the self-management group program. Finally, we used the Kihon checklist to define robust, pre-frail, and frail older adults. The frailty screening index based on the Cardiovascular Health Study criteria is widely used for frailty assessment worldwide [2]. Satake et al. reported that the frail category by the Kihon checklist is validated by the Cardiovascular Health Study criteria [9]. Therefore, these results should be interpreted with caution.

This is the first study to demonstrate that a community-based self-management group is effective in preventing new LTCI service requirement certifications during a four-year follow-up period in community-dwelling older adults. These results suggest the usefulness of self-management group activities to reduce disability incidence in older adults. We therefore recommend that self-management groups be widely established in each community.

## 5. Conclusions

The current study found that community-based self-management group exercise reduced the incidence of disability during a four-year follow-up period in community-dwelling older adults. Thus, increasing self-management group activities in each community should be encouraged.

## Figures and Tables

**Table 1:** Baseline characteristics of the study participants in the two groups.

Baseline Characteristics		Participation Group		Control Group		*p*-Value
		Mean	SD	Mean	SD	
Overall		n = 1620		n = 1620		
Age	year	77.1	6.4	77.2	6.9	0.77
Height	cm	153.8	8.8	153.8	9.1	0.89
Weight	kg	53.0	9.8	52.8	10.2	0.68
BMI		22.4	3.5	22.3	3.9	0.67
Gender	women, n (%)	1330	82.1%	1336	82.5%	0.78
KCL	scores	5.3	3.8	5.3	3.7	0.68
Robust		n = 609		n = 609		
Age	year	75.3	5.9	75.2	6.0	0.81
Height	cm	154.4	8.6	155.1	8.6	0.13
Weight	kg	53.7	9.4	54.3	9.6	0.29
BMI		22.5	3.3	22.5	3.6	0.80
Gender	women, n (%)	482	78.8%	480	79.1%	0.89
KCL	scores	1.8	1.0	1.7	1.1	0.40
Pre-frail		n = 591		n = 591		
Age	year	77.5	6.4	77.4	6.8	0.72
Height	cm	154.0	9.0	154.0	9.3	0.93
Weight	kg	53.0	10.2	52.8	10.5	0.67
BMI		22.3	3.6	22.3	4.1	0.83
Gender	women, n (%)	491	83.1%	498	84.3%	0.58
KCL	scores	5.3	1.1	5.3	1.1	0.77
Frail		n = 420		n = 420		
Age	year	79.2	6.5	80.0	7.4	0.10
Height	cm	152.6	8.8	151.7	9.2	0.17
Weight	kg	51.8	9.6	50.8	10.1	0.13
BMI		22.2	3.7	22.0	3.9	0.39
Gender	women, n (%)	357	85.0%	358	85.2%	0.92
KCL	scores	10.5	2.5	11.0	2.8	0.53

BMI: body mass index, KCL: Kihon checklist, SD: standard deviation.

**Table 2:** Cox proportional hazards models of each group for new LTCI service requirement certifications during the two- or four-year follow-up period.

**Two-Year Follow-Up**								
**Frailty Status/Group**		**New LTCI/Total Number (%)**		**HR**	**95% CI**			***p*-Value**
					**Min**	**-**	**Max**	
Overall	P-group	155/1620	9.6%	1.00	ref			
	C-group	155/1620	9.6%	1.01	0.81	-	1.26	0.947
Robust	P-group	16/609	2.6%	1.00	ref			
	C-group	18/609	3.0%	1.13	0.58	-	2.21	0.725
Pre-frail	P-group	44/591	7.4%	1.00	ref			
	C-group	52/591	8.8%	1.20	0.80	-	1.79	0.370
Frail	P-group	95/420	22.6%	1.00	ref			
	C-group	85/420	20.2%	0.90	0.67	-	1.20	0.465
**Four-Year Follow-Up**								
**Frailty Status/Group**		**New LTCI/Total Number (%)**		**HR**	**95% CI**			***p*-Value**
					**Min**	**-**	**Max**	
Overall	P-group	334/1620	20.6%	1.00	ref			
	C-group	247/1620	15.2%	0.73	0.62	-	0.86	<0.001
Robust	P-group	40/609	6.6%	1.00	ref			
	C-group	32/609	5.3%	0.80	0.50	-	1.27	0.343
Pre-frail	P-group	117/591	19.8%	1.00	ref			
	C-group	95/591	16.1%	0.81	0.62	-	1.06	0.121
Frail	P-group	177/420	42.1%	1.00	ref			
	C-group	120/420	28.6%	0.65	0.51	-	0.82	<0.001

P-group: participation group, C-group: control group, LTCI: long-term care insurance, HR: hazard ratio, CI: confidence interval, Min: minimum, Max: maximum.
